# Measuring Staff Empowerment Regarding Health Care for Clients with Intellectual Disabilities

**DOI:** 10.1155/2014/678127

**Published:** 2014-02-25

**Authors:** Joanne Wilkinson, Nechama W. Greenwood, Claire Tienwey Wang, Laura F. White, Larry Culpepper

**Affiliations:** ^1^Department of Family Medicine, Boston University School of Medicine, Dowling 5, 771 Albany Street, Boston, MA 02118, USA; ^2^Department of Community Health Sciences, Boston University School of Public Health, 801 Massachusetts Avenue, Boston, MA 02118, USA; ^3^Department of Biostatistics, Boston University School of Public Health, 715 Albany Street, Boston, MA 02118, USA

## Abstract

*Background*. Women with intellectual disabilities (ID) contract breast cancer at the same rate as the general population but have higher breast cancer mortality and lower rates of breast cancer screening. Many women with ID live in group homes or supported residences where they are cared for by direct support workers. While direct support workers are thought to influence client health, this effect is underresearched, and we lack tools for measuring staff empowerment and perceptions regarding client health. *Methods*. We developed and validated an instrument, the staff empowerment tool (SET), to measure staff empowerment as related to supporting clients in preventive health. *Results*. The SET was found to be a reliable instrument for measuring staff activation and empowerment in helping clients access mammography screening. *Discussion*. Quantifying staff empowerment and perspectives is important in studying and reducing disparities among adults with ID, a vulnerable population. Further research to determine the impact of staff empowerment levels on their clients' health and health care access is suggested. The SET is a valuable tool for measuring the construct of staff empowerment, evaluating interventions, and collecting data regarding variation in staff empowerment.

## 1. Introduction

Adults with intellectual disabilities (ID) are living longer [1] and experiencing multiple age-related health disparities that have been well-documented [2–4]. One such disparity is low rates of age-appropriate screenings [4–6], particularly mammography for women [7]. Women with ID are diagnosed with breast cancer at similar rates to women in the general population [8, 9] yet have low rates of mammography, under 50% in many studies [10, 11]. It is important for policy makers, clinicians, and researchers to better understand the reasons for this disparity in order to improve screening rates; also, factors affecting mammography may also affect other age-appropriate screenings and health disparities among adults with ID.

One factor which probably affects the mammography rate, yet is poorly understood, is the impact of staff members or direct support workers on the health behaviors of women with ID who live in group homes [12]. In a large administrative database analysis of women with ID in Massachusetts, it was noted that clients with ID who lived in 24-hour residential settings were more likely to receive mammograms than clients who lived with family [13], an effect most pronounced in residential settings with an RN to coordinate care. Another study based in the Midwest [14] noted the low health literacy of the average direct support worker. Qualitative studies with physicians [15] and women with ID [16, 17] both revealed that women may be accompanied for health appointments by a staff member who does not know them well. The role of direct support workers and staff in empowering and implementing health decisions such as screening for their clients is potentially great. However, little is known about staff members' own empowerment and knowledge regarding their clients' health. Given that roughly 35% of women with ID live in 24-hour residential settings [13, 16], it is important to consider the potentially powerful influence of staff on screening behaviors in this population.

Empowering staff to help engage women with ID in breast cancer screening seems potentially helpful and important, yet there are scant data regarding the idea of empowerment in staff members or how it should be measured. The ideas of patient empowerment and “activation” [18] have been studied by many researchers. In many studies, interventions that increase patients' activation or empowerment have been shown to result in increased ability to self-manage chronic disease, even in patient groups that had previously experienced disparities [19]. However, it was also noted in a different study that high levels of patient activation were only correlated with some illness management behaviors and not others [20]. Particularly for patients with intellectual disabilities, this raises the question of who should be empowered: the patient, the support worker/family member, or both. There is some precedent for conceptualizing empowerment as a shared entity between patient and family member in pediatric [21] and geriatric patient populations, and for simply aiming to empower everyone involved in the health encounter with the goal of making the healthcare environment more collaborative and less paternalistic [22]. Finally, some researchers have begun to explore the idea of empowerment for patients with intellectual disabilities [23], though the theorized role of staff in empowering the patients is unclear [24].

We became interested in staff members' activation and empowerment as a result of our work on two previous qualitative studies focused on mammography in women with ID. In the first study 26 women with ID were interviewed regarding their experiences, barriers and facilitators of mammography. In the second study, 22 physicians were interviewed regarding health decisions for people with ID. While these data were published relative to mammography and physicians' educational needs [13, 15, 16], both women with ID and physicians who care for them mentioned staff members as an important factor in health care for women with ID. Measuring both staff members' actual knowledge about mammograms and the degree to which they feel empowered to help their clients attain better health is an important step toward understanding and potentially improving the influence of staff members on the health behaviors (specifically mammography) of women with ID. We developed a tool called the Staff Empowerment Tool or the SET. We then used the SET to collect preliminary data on staff empowerment regarding mammography screening, which will be reported here.

## 2. Methods

To develop and collect preliminary data using the SET, we used a multistep process. To inform the development of the instrument we conducted a targeted literature review looking for similar instruments and concepts; using the derived information we then designed and conducted focus groups and individual qualitative interviews to define important realms of empowerment and knowledge. We then collected pilot data using cognitive interviewing to solicit feedback regarding the clarity and utility of the tool. This feedback was used to clarify previously confusing wording. Finally we used the revised instrument to collect data from larger groups of staff members in multiple settings in two states.

### 2.1. Literature Review

We used PubMed and Google Scholar search engines to locate studies containing one or more of the following key words: “intellectual disability,” “staff,” “patient empowerment,” “patient activation,” “shared decision making,” “paid care giver,” “caregiver,” and “group home.” We reviewed current concepts regarding patient empowerment and “activation” (see above), with special attention paid to studies related to patient empowerment in the context of relationships, such as parental empowerment on behalf of ill children, or activation of the children of elderly adults with dementia. Based on our prior work in health promotion with people with intellectual disabilities, we theorized that it was important to conceptualize empowerment using the ecological model of health behavior [25] for people with intellectual disabilities. In brief, the ecological model posits that health decisions are not made in a vacuum but rather involve the input of the patient and multiple levels or layers of the environment around them: interpersonal, social, community, and population level influences. We decided to focus our instrument development at the interpersonal and community levels, where the influence of a staff member would be felt. We concluded that there was not an appropriate preexisting instrument to measure staff empowerment, though there were elements of some of the patient measures that could also apply to staff members. We therefore decided to proceed with qualitative research to develop and pilot a new instrument.

### 2.2. Qualitative Data

We then revisited data from two prior qualitative studies we had previously conducted. This data included interviews with 26 women with ID and 22 physicians regarding health decisions for people with ID. While these data were published relative to mammography and physicians' educational needs [13, 15, 16], the original transcripts were reanalyzed looking for themes related to staff behaviors that impeded or reinforced mammography using content analysis to identify quotes related to the impacts of staff on health beliefs, behaviors, and experiences. We first used deductive content analysis, a qualitative method where prior knowledge of the topic is used to theorize concepts of importance [26], to identify appropriate passages for analysis. We then used grounded coded theory [27], an inductive qualitative method where theories are generated from the data, to identify themes related to staff and health. Emerging themes were used to develop and refine a code book which was used by all researchers. Each transcript was discussed by at least two coders, in order to identify and reconcile intercoder differences.

We also conducted three focus groups: one with women with ID (*n* = 4), one with staff only (*n* = 5), and one mixed group (*n* = 6). Participants for these focus groups were recruited by posting an advertisement with a community agency and using snowball sampling from the women in the original qualitative study. The focus group was used to triangulate data from the reanalysis of the previous transcripts and to verify that we had identified useful constructs. All of the women with ID were 40 or over and able to understand the study and give their consent. In a few cases, women had guardians who were also approached for formal consent. Though this research design with individuals with ID may exclude those who lack the functional ability to provide informed consent or participate in a focus group, we selected this design in order to maintain ethical standards of research. The semi-structured interview guide for the staff focus group was as follows.

- Can you tell us about your experiences taking your client to the doctor? What has gone well and what hasn't gone so well?
- Have you ever taken a client to a mammogram? How was it? Were there things that would have helped you to know ahead of time?
- If you were helping train a new staff member who had never taken a client to the doctor, what would you tell them? What about for taking clients to mammograms—what would you tell.
- Thinking about yourself and the people you work with, what do you think are the biggest challenges to being successful in taking clients for medical appointments? What would help.

Data from the focus groups were analyzed inductively with grounded coded theory and deductively with content analysis, using the code book developed during our transcript review.

### 2.3. Instrument Development and Pilot Data Collection

We developed a draft of the SET using items similar to those in existing instruments to measure patient activation and health locus of control [18, 28], which our literature search and qualitative analysis had identified as most important. After relevant constructs were identified through these methods, we looked for validated instruments measuring the constructs, which were then adapted to the population. The SET was then pilot tested with ten staff members using cognitive interviewing, a technique where participants are asked to provide answers and then it explains the thinking behind their answers [29]. Data from cognitive interviewing was used to refine the survey instrument, a common practice in survey design. For example, our cognitive interviewing showed items that were worded in a confusing manner, allowing us to reword potentially confusion survey items. The staff for the pilot data collection all worked at two agencies in Massachusetts who were familiar with our community work but had not participated in the initial qualitative studies. Based on their feedback, a few items were removed and the wording was changed in others. The final instrument is shown in Table 1.

### 2.4. Data Collection and Analysis

In order to test the instrument we developed as described above, participants, direct support workers employed in group home settings serving clients with ID, were recruited through a number of agencies providing residential services to people with ID in Massachusetts, Alabama, and Tennessee. (These three settings were chosen to ensure diversity in the sample, since Massachusetts currently has universal health insurance and because our team was preparing to do an intervention in Alabama and Tennessee and was collecting pilot data.) The SET was distributed at staff meetings with a consent form and staff members who did not wish to participate simply did not fill it out. In total, 3 agencies were approached with a yield of 61 completed SETs.

We used the data to calculate a mean and standard deviation for the entire instrument and for the mammography-specific questions. We also compared the scores on general health-related items with the scores on mammography items using *t*-tests and, for a subsample of the population for whom we had demographic information, examined mean scores on the SET by years of experience as a support worker. Finally, we conducted a confirmatory factor analysis using a polychoric correlation matrix to create composite factor scores based on the hypothesized groupings of the questions in the survey instrument. As a sensitivity analysis, we also performed a principal components analysis to determine if the hypothesized groupings were supported by the data. All analyses were performed using SAS v. 9.3 (SAS Institute, Cary, NC).

## 3. Results

**Figure 1:**
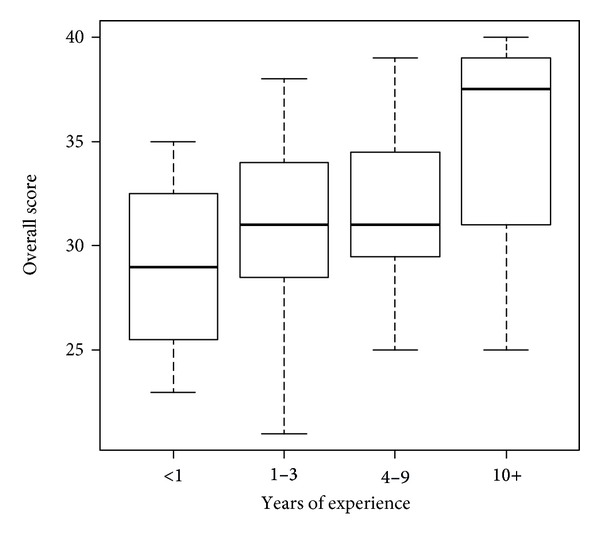
Years of experience versus total score on SET trend.

The focus group data effectively triangulated data from the existing qualitative data, suggesting that the SET instrument evaluates important constructs in terms of staff empowerment regarding client health.  Table 2 shows the median and range of the scores for each item and the sum of the items. Principal components analysis confirmed our hypothesized groupings of the questions in the survey instrument, creating two distinct clusters of questions. The first three questions loaded on the same factor and questions 4–8 loaded on another distinct factor. Using the scores we generated for each of the factors, we found that the two factors were weakly correlated with each other (Spearman correlation = 0.04, *P* = 0.76). Additionally we found that as the years of job experience of the staff member increased (see Figure 1), there was a significant increasing trend in the overall score (*P* = 0.05).

## 4. Discussion

The SET is a new instrument we developed to quantify and measure staff empowerment to facilitate mammography in their clients with intellectual disability. Other studies have suggested that the impact of staff members, particularly for patients living in 24-hour residential settings in the community, is probably significant but difficult to measure [12, 13, 16]. The SET can be used to assess the level of staff empowerment to impact the health of their clients. This is different from simple health knowledge, and reflects the perception of the staff member of their own potential effectiveness at positively impacting clients' health. While these data are preliminary, we believe the SET has potential to reflect a concept which to date has been unmeasured in studies of mammography disparities in women with ID. The SET is not adjusted for staff member literacy or health literacy levels and does not measure the staff members' personal beliefs or health behaviors. As such, it is a practical, client-centered instrument that focuses on staff members' interactions with their clients with ID.

Our analysis showed that staff members' SET scores were positively correlated with years of experience working with people with ID. Research shows that increased exposure to the population of people with ID increases comfort with the population [30, 31] This suggests that empowerment regarding client health may be positively correlated with increased comfort with the population. Further research is needed, however, to more fully explain this correlation.

Other researchers have commented on the importance of involving staff in the health of clients with ID and somehow being able to measure or assess their impact [32]. With regard to increasing rates of mammography for women with ID, this measurement seems important in terms of understanding where to focus efforts at education and empowerment in future studies [33, 34]. The SET provides a means of measuring a staff empowerment regarding their clients' health, a construct that impacts health outcomes for people with ID, but has not been quantitatively measured before.

This study has several limitations. Firstly, the SET was developed using data from our previous studies, which allowed us to access over one hundred single spaced pages of transcripts, representing a wealth of data. This may also be a source of bias, in that this may have affected the scope of the study and/or the instrument development process. Conversely, this enabled us to benefit from expertise developed over the course of several years. In addition, constructs used in the SET were evaluated in focus groups that included staff members and women with intellectual disabilities. The focus groups only included women with ID who had sufficient verbal and functional skills to provide informed consent and meaningful participation, a potential limitation in that women with more severe forms of ID were excluded due to inability to participate. While women with more severe ID may have additional barriers or concerns that would not be addressed by their less severely impacted counterparts, we theorize that they would be impacted by the same factors as women with milder ID. In this sense, women with milder ID acted as proxies for their more severely disabled peers, who might be more negatively impacted by the inequalities and barriers identified by our participants. The SET was tested using agencies that volunteered to recruit their staff; while staff members had the option not to complete the instrument, it is possible that these agencies already had a higher-than-usual level of staff involvement regarding clients' health because of their interest in the study. While the SET was tested in three states (Massachusetts, Tennessee, and Alabama), the sample was by no means a comprehensive reflection of staff in residential settings throughout the US.

Staff members' attitudes and level of empowerment regarding their clients' health are thought to impact the health of people with intellectual disabilities, a known disparity population. Previously, we lacked the means to measure staff empowerment. Without a means of measuring this construct, we are unable to quantify the impact of staff attitudes on client health or to evaluate the impact of future interventions to increase staff activation. Though the SET has some limitations, we hope that it can be used to evaluate interventions and to collect data regarding regional and other differences in staff activation. The SET might also have utility in studies evaluating the impact of staff training, organizational culture change, and health promotion interventions that target care givers of adults with ID. Though the SET is focused on mammography, we hope that future researchers can use a similar process to adapt mammography-specific SET items to evaluate empowerment regarding other health issues in adults with ID, such as obesity prevention, other screening tests, such as colonoscopy, and primary health topics. We welcome future research regarding the applicability of the SET to other health topics, and we are currently developing a version of the SET related to staff members' empowerment regarding chronic illness management in patients with severe mental health disorders. We hope that others will use this instrument, or an adapted version of it, as described above in their own work to improve health disparities for people with ID. This would also allow the SET to eventually be used and assessed in a variety of diverse geographic and demographic settings.

## Figures and Tables

**Table 1:** Final version of Staff Empowerment Tool (SET).

It is important that I take an active role in my client's health.	1	2	3	4	5
I have an impact on my client's health.	1	2	3	4	5
I know how to help my client prevent problems with her health.	1	2	3	4	5
I know how often my client should have a mammogram and when she is due for a mammogram.	1	2	3	4	5
If my client asked me to help her schedule a mammogram, I would know how to help her.	1	2	3	4	5
I am confident that I can support my client during a mammogram.	1	2	3	4	5
I am confident I can help my client understand what will happen during her mammogram.	1	2	3	4	5
I can describe what a mammogram does and why it is important.	1	2	3	4	5

1: strongly disagree; 2: disagree; 3: neither agree nor disagree; 4: agree; 5: strongly agree.

**Table 2:** Per-question and total mean scores and standard deviation of SET.

	Median (range)
It is important that I take an active role in my client's health.	5.0 (3–5)
I have an impact on my client's health.	4 (2–5)
I know how to help my client prevent problems with her health	4 (2–5)
I know how often my client should have a mammogram and when she is due for a mammogram.	3 (2–5)
If my client asked me to help her schedule a mammogram, I would know how to help her.	4 (1–5)
I am confident that I can support my client during a mammogram.	4 (1–5)
I am confident I can help my client understand what will happen during her mammogram.	4 (1–5)
I can describe what a mammogram does and why it is important.	4 (1–5)

Total	30 (18–40)
